# Breakfast Skipping and Elevated Neck Circumference Are Independently Associated with Newly Diagnosed Dyslipidemia in Adults Without Diabetes

**DOI:** 10.3390/jcm15103734

**Published:** 2026-05-13

**Authors:** Nezihe Otay Lule, Kemal Ozan Lule, Ozge Ozsoy, Hamit Yildiz

**Affiliations:** 1 Nutrition and Dietetics Department, Faculty of Health Sciences, Gaziantep University, 27310 Gaziantep, Türkiye; 2 Internal Medicine Department, Faculty of Medicine, Gaziantep University, 27310 Gaziantep, Türkiye; drkemalozanlule@gmail.com (K.O.L.); drhyildiz@hotmail.com (H.Y.); 3 Internal Medicine Department, Araban District State Hospital, 27650 Gaziantep, Türkiye; ozsoyozgee@gmail.com

**Keywords:** dyslipidemia, breakfast skipping, neck circumference, meal pattern, anthropometric risk, cardiometabolic risk, waist-to-height ratio, lipid profile

## Abstract

**Background/Objectives:** Dyslipidemia is a major cardiometabolic disorder frequently accompanied by adverse fat distribution and unhealthy eating behaviors. This study aimed to compare meal pattern characteristics and anthropometric indicators between adults with newly diagnosed dyslipidemia and normolipidemic controls and to identify factors independently associated with dyslipidemia. We hypothesized that breakfast skipping would be more prevalent among individuals with dyslipidemia and that distribution-based anthropometric markers—particularly neck circumference (NC) and waist circumference (WC)—would be more strongly associated with dyslipidemia than body mass index (BMI). **Methods:** This cross-sectional comparative study included 257 adults without diabetes aged 18–65 years. Anthropometric assessment included BMI, WC, waist-to-height ratio (WHtR), and NC. Meal patterns were assessed using a structured questionnaire. Hierarchical binary logistic regression was used to identify independent associations. The linearity assumption was verified, and a sensitivity analysis was performed with HbA1c dichotomized at ≥5.7%. **Results:** Elevated NC was more frequent in participants with dyslipidemia (95.6% vs. 78.3%, *p* < 0.001). Breakfast skipping was more common among participants with dyslipidemia (30.7% vs. 15.0%, *p* = 0.003), whereas lunch skipping was less common (52.6% vs. 65.8%, *p* = 0.031). In the fully adjusted model, elevated NC (OR: 4.72), breakfast skipping (OR: 3.39), and HbA1c (OR: 3.61) were independently associated with dyslipidemia. These findings were confirmed in sensitivity analysis. **Conclusions:** Breakfast skipping and elevated NC were independently associated with newly diagnosed dyslipidemia, partially supporting the study hypothesis. Breakfast skipping and NC may aid in early risk identification in outpatient settings. Prospective studies are needed to confirm these associations.

## 1. Introduction

Dyslipidemia is a major metabolic disorder that plays a central role in the development of cardiovascular disease and frequently co-occurs with obesity and central adiposity [1]. Although the management of dyslipidemia has traditionally focused on lipid-lowering targets, increasing attention has been directed to the role of lifestyle behaviors—particularly, meal patterns and meal skipping habits—on lipid metabolism and anthropometric risk indicators [2]. Dietary behaviors such as meal timing and frequency have been proposed to affect weight regulation, insulin resistance, free fatty acid mobilization, lipoprotein metabolism, and overall cardiometabolic health [3,4,5,6]. Across cohort studies, habitual breakfast skipping and late-night eating have been consistently associated with increased risk of dyslipidemia and systemic inflammation [7]. These associations are increasingly understood within the framework of chrononutrition, which highlights that misalignment between meal timing and endogenous circadian rhythms can impair lipid metabolism, reduce insulin sensitivity, and promote adverse cardiometabolic profiles [8]. In particular, breakfast skipping has been linked to unfavorable lipid changes in both cross-sectional and prospective designs, and a systematic review of randomized controlled trials and cohort studies reported that habitual breakfast omission was associated with higher low-density lipoprotein (LDL) cholesterol levels [9]. More broadly, an umbrella review of systematic reviews confirmed that meal timing and frequency are associated with alterations in lipid profiles, although the magnitude and direction of these associations vary across study populations and dietary contexts [10]. Notably, most available evidence comes from general population samples that often include individuals with pre-existing metabolic conditions or ongoing pharmacotherapy, limiting the ability to isolate behavioral correlates of dyslipidemia at the time of initial diagnosis.

Despite this growing evidence base, studies specifically examining the relationship between meal skipping behaviors and dyslipidemia remain limited, and many existing investigations are complicated by the inclusion of individuals with diabetes mellitus, lipid-lowering medication use, or advanced metabolic comorbidities, all of which may substantially influence lipid profiles and related metabolic parameters [11,12]. Evaluating newly diagnosed individuals before long-term treatment exposure may therefore provide a clearer picture of behavioral and anthropometric correlates present at the time dyslipidemia is first identified. In parallel, anthropometric indicators such as waist circumference (WC), waist-to-height ratio (WHtR), neck circumference (NC), and body mass index (BMI) are widely used in cardiometabolic risk assessment; however, these measures may not capture risk equally well. WC is an established surrogate for visceral adiposity and has been endorsed as a vital clinical measurement by international consensus [13]; however, its discriminative value may be attenuated in populations with high overall adiposity, where most individuals already exceed standard clinical thresholds. WHtR has been proposed as a height-adjusted alternative that may improve cardiometabolic risk stratification [14], and prospective data suggest it may predict incident dyslipidemia independently of BMI [15]; however, evidence specifically linking WHtR to individual dyslipidemia components remains limited. In contrast, NC, considered a marker of upper-body subcutaneous adiposity, has attracted increasing interest as a simple, practical, and potentially informative indicator of cardiometabolic risk that may capture metabolic variation not fully reflected by WC or BMI [16]. Nevertheless, studies jointly evaluating meal-skipping behaviors and multiple anthropometric risk indicators—including NC, WC, and WHtR—in newly diagnosed, treatment-naïve, adults without diabetes with dyslipidemia are lacking. This gap is important because examining individuals before the initiation of lipid-lowering therapy or the development of advanced comorbidities may allow a more accurate assessment of the behavioral and anthropometric correlates present when dyslipidemia is first identified.

This study aimed to compare meal pattern characteristics and anthropometric risk indicators between adults with newly diagnosed dyslipidemia and normolipidemic controls, and to identify factors independently associated with dyslipidemia using hierarchical logistic regression analysis. Based on prior evidence, we hypothesized that breakfast skipping would be more prevalent among dyslipidemic individuals and that anthropometric markers of fat distribution—particularly NC, WC, and WHtR —would be more strongly associated with dyslipidemia than conventional obesity metrics such as BMI.

## 2. Materials and Methods

### 2.1. Study Design and Population

This cross-sectional comparative study was conducted at the General Internal Medicine Outpatient Clinic of Gaziantep University Şahinbey Research and Practice Hospital between February 2026 and March 2026. The study population consisted of adults aged 18–65 years who attended the outpatient clinic and underwent routine fasting lipid profile assessment during the study period.

Participants were classified into two groups based on fasting lipid profile results obtained during routine clinical evaluation:

- Normolipidemia group: Individuals who did not meet any of the predefined dyslipidemia criteria and had no history of dyslipidemia.
- Dyslipidemia group: Individuals who met at least one of the predefined dyslipidemia criteria and had no previous history of dyslipidemia.

Dyslipidemia was defined as the presence of at least one abnormal lipid parameter based on commonly used clinical thresholds (triglycerides ≥ 150 mg/dL, LDL cholesterol ≥ 130 mg/dL, HDL cholesterol < 40 mg/dL in men or <50 mg/dL in women) [17,18,19].

Consecutive eligible participants were recruited during routine outpatient visits. The study compared meal pattern characteristics and anthropometric risk indicators between individuals with newly diagnosed dyslipidemia and normolipidemic controls.

Sociodemographic characteristics (age and sex), anthropometric measurements (height, body weight, WC, and NC), meal pattern characteristics (number of main meals and snacks per day, and skipping of breakfast, lunch, dinner, and snacks), and biochemical parameters obtained during routine clinical evaluation (triglycerides, LDL cholesterol, HDL cholesterol, fasting glucose, and HbA1c) were collected.

Biochemical parameters were obtained from routine outpatient laboratory measurements. No additional laboratory tests were requested specifically for the purposes of this study.

Anthropometric measurements and questionnaire-based meal pattern data were collected prospectively from participants who provided informed consent.

### 2.2. Inclusion and Exclusion Criteria

Participants were eligible for inclusion if they met the following criteria:

1. Age between 18 and 65 years;
2. Newly diagnosed dyslipidemia (case group) or normolipidemia (control group);
3. Availability of fasting lipid profile (triglycerides, LDL, HDL), fasting glucose, and HbA1c measurements obtained as part of routine clinical care;
4. Adequate cognitive and communication ability to complete the questionnaire;
5. Willingness to participate in the study.

Participants were excluded if they met any of the following criteria:

1. Age < 18 or >65 years;
2. Diagnosis of diabetes mellitus (HbA1c ≥ 6.5% or known history of diabetes);
3. Previous use of lipid-lowering medications;
4. Presence of a known physician-diagnosed chronic inflammatory, endocrine, or malignant disease;
5. Missing data for key study variables;
6. Pregnancy or lactation.

Participants attending the outpatient clinic during the study period were screened for eligibility. Exclusion criteria were applied independently and were not mutually exclusive; therefore, a single individual could meet more than one exclusion criterion. The number of participants excluded for each criterion is presented in the participant flow diagram (Supplementary Figure S1). Missing data were limited to anthropometric circumference measurements in 13 participants whose measurements could not be obtained during routine outpatient visits; this missingness was considered random and unrelated to dyslipidemia status. A total of 257 participants met all inclusion criteria and were included in the final analysis (120 normolipidemic, 137 dyslipidemic).

### 2.3. Sample Size and Power Analysis

Sample size was calculated using G*Power 3.1 software based on previously reported effect sizes for the association between meal frequency and lipid parameters [20]. Assuming an effect size of d = 0.40, a two-sided α = 0.05, and 80% power for comparisons between two independent groups, a minimum of 100 participants per group was required (two-sample *t*-test). Allowing for approximately 10% data loss, the target sample size was set at 220 participants (110 cases and 110 controls).

### 2.4. Data Collection

Data were collected using a structured questionnaire developed by the researchers specifically for this study. The questionnaire comprised closed-ended, factual items and was reviewed for content clarity by all members of the research team prior to data collection. Because the items assessed straightforward behavioral information rather than latent constructs, formal psychometric evaluation was not undertaken.

The questionnaire included:
Sociodemographic characteristics (age and sex)
Meal pattern characteristics:
  - Number of main meals per day;
  - Number of snacks per day;
  - Skipping of breakfast, lunch, dinner, and snacks (Supplementary File S1).

Meal skipping was assessed using a three-category response format (always skip, sometimes skip, never skip). For analytical purposes, participants who responded “always skip” or “sometimes skip” were classified as meal skippers, whereas those who responded “never skip” were classified as non-skippers.

### 2.5. Anthropometric Measurements

All anthropometric measurements (height, body weight, WC, NC) were performed by a single trained researcher according to standardized protocols. The assessor was blinded to participants’ lipid profile results at the time of measurement. Height was measured using a stadiometer with participants standing in the Frankfurt plane. Body weight was measured to the nearest 0.1 kg using a calibrated digital scale. Body mass index (BMI) was calculated as body weight (kg) divided by height squared (m^2^) [21]. WC was measured at the midpoint between the lowest rib and the iliac crest. WC values were classified as ≥94 cm (risk) and ≥102 cm (high risk) in men and ≥80 cm (risk) and ≥88 cm (high risk) in women [22]. WHtR was calculated as WC divided by height. Values ≥ 0.5 were considered increased health risk and ≥0.6 markedly increased risk [23]. NC was measured using a non-elastic measuring tape just below the cricothyroid cartilage with the head positioned in the Frankfurt plane. Values ≥ 37 cm in men and ≥34 cm in women were classified as elevated NC according to sex-specific cutoff values [24].

### 2.6. Laboratory Measurements

Triglycerides, LDL cholesterol, HDL cholesterol, fasting plasma glucose, and HbA1c values were obtained from routine outpatient biochemical analyses performed at the time of clinic admission. No laboratory tests were requested specifically for research purposes. To quantify cumulative lipid abnormality burden, a dyslipidemia component count variable was constructed. This variable represented the number of abnormal lipid parameters (elevated LDL cholesterol, elevated triglycerides, and reduced HDL cholesterol) and ranged from 0 to 3. This composite variable was constructed for the purposes of the present study and has not been independently validated as a clinical instrument; it was used descriptively to quantify cumulative lipid abnormality burden across anthropometric risk categories.

### 2.7. Statistical Analysis

All statistical analyses were performed using IBM SPSS Statistics software (version 24.0; IBM Corp., Armonk, NY, USA). A two-sided *p* value < 0.05 was considered statistically significant. The distribution of continuous variables was assessed using graphical (histograms and probability plots) and analytical methods. Normally distributed variables were presented as mean ± standard deviation, whereas non-normally distributed variables were expressed as median (interquartile range). Categorical variables were presented as number and percentage. Comparisons between participants with and without dyslipidemia were performed using the independent samples *t*-test for normally distributed variables and the Mann–Whitney U test for non-normally distributed variables. The chi-square test was used for categorical variables.

Comparisons of lipid parameters (triglycerides, HDL cholesterol, LDL cholesterol) and dyslipidemia component count across anthropometric risk categories (NC risk status, WC risk status, and WHtR risk categories) were performed using independent samples *t*-test or one-way ANOVA for normally distributed variables and the Kruskal–Wallis test for non-normally distributed variables.

Independent factors associated with dyslipidemia were evaluated using hierarchical binary logistic regression analysis, with dyslipidemia status (yes/no) as the dependent variable. Variables were entered sequentially according to predefined conceptual domains.

- Model 1 (Demographic model) included age and sex.
- Model 2 (Anthropometric model) additionally included NC risk status and WC risk status. For regression analyses, WC categories were dichotomized as no risk versus any risk present.
- Model 3 (Behavioral model) additionally included breakfast skipping status.
- Model 4 (glycemic-adjusted exploratory model) additionally included HbA1c, because glycemic status may cluster with lipid abnormalities even in individuals without diagnosed diabetes; this variable was added to assess the robustness of the main associations after further adjustment for subclinical glycemic variation.

Anthropometric risk variables were entered as binary variables (presence vs. absence of risk), and breakfast skipping was coded as skip (always or sometimes) versus never skip. Results were expressed as odds ratios (ORs) with 95% confidence intervals (CIs). Model fit was evaluated using the likelihood ratio chi-square test (Omnibus χ^2^), explanatory power using Nagelkerke R^2^, and calibration using the Hosmer–Lemeshow goodness-of-fit test. Multicollinearity was assessed using variance inflation factors (VIFs) obtained from an equivalent linear regression model, and no evidence of multicollinearity was observed (all VIF values < 2). The linearity assumption for continuous variables in logistic regression was assessed using the Box-Tidwell test. Age satisfied the linearity assumption (*p* = 0.234), whereas HbA1c did not (*p* < 0.001). Therefore, in a sensitivity analysis, HbA1c was dichotomized using the established prediabetes threshold (≥5.7%) according to current diagnostic standards [25]. No significant interaction was observed between elevated NC and sex (*p* = 0.563); therefore, no interaction terms were retained in the final models.

## 3. Results

### 3.1. Clinical and Anthropometric Characteristics of the Study Groups

The clinical and anthropometric characteristics of the normolipidemic and dyslipidemic participants are presented in Table 1. A total of 257 participants were included, including 120 normolipidemic and 137 dyslipidemic individuals. Age, sex distribution, body mass index, and elevated WC were comparable between groups (all *p* > 0.05). In contrast, the dyslipidemic group had significantly higher fasting glucose and HbA1c levels than the normolipidemic group (all *p* < 0.001). A higher proportion of participants in the dyslipidemic group had elevated NC than in the normolipidemic group (95.6% vs. 78.3%, *p* < 0.001). Although mean WHtR did not differ significantly between groups (*p* = 0.094), WHtR category distribution differed significantly (*p* = 0.048), with a higher proportion of high-risk individuals among dyslipidemic participants.

### 3.2. Meal-Skipping Characteristics According to Dyslipidemia Status

The meal-skipping characteristics of the normolipidemic and dyslipidemic participants are presented in Table 2. The mean number of main meals and snacks per day did not differ significantly between the groups (both *p* > 0.05). However, meal-skipping patterns differed significantly between the groups. Breakfast skipping was more frequent among dyslipidemic participants than among normolipidemic participants (30.7% vs. 15.0%, *p* = 0.003), whereas lunch skipping was less common in the dyslipidemic group (52.6% vs. 65.8%, *p* = 0.031). No significant differences were observed for dinner skipping or snack skipping (both *p* > 0.05).

### 3.3. Association of Anthropometric Indicators with Dyslipidemia Burden and Lipid Profile

Table 3 presents dyslipidemia component burden and lipid parameters across anthropometric indicators. Participants with elevated neck circumference had a significantly greater dyslipidemia component count and a less favorable lipid profile, with lower HDL and higher LDL levels, than those without elevated neck circumference (all *p* ≤ 0.003), whereas triglyceride levels did not differ significantly (*p* = 0.063). Similarly, participants with elevated waist circumference had a higher dyslipidemia component count and lower HDL levels than those without elevated waist circumference (*p* = 0.023 and *p* = 0.030, respectively), while triglyceride and LDL levels were similar between the groups. Across waist-to-height ratio categories, the overall difference in dyslipidemia component count approached statistical significance (*p* = 0.051), whereas triglyceride, HDL, and LDL levels did not differ significantly.

### 3.4. Hierarchical Logistic Regression Analysis for Dyslipidemia

Table 4 presents the hierarchical logistic regression models for dyslipidemia. In the demographic model (Model 1), neither age nor sex was significantly associated with dyslipidemia. After the inclusion of anthropometric variables (Model 2), elevated neck circumference emerged as an independent factor associated with dyslipidemia (OR: 5.14, 95% CI: 1.94–13.60, *p* = 0.001), whereas elevated waist circumference was not significant. In the behavioral model (Model 3), both elevated neck circumference (OR: 6.03, 95% CI: 2.18–16.65, *p* = 0.001) and breakfast skipping (OR: 3.21, 95% CI: 1.62–6.37, *p* = 0.001) were independently associated with dyslipidemia. In the final exploratory model additionally adjusted for HbA1c, elevated neck circumference, breakfast skipping, and HbA1c remained independently associated with dyslipidemia. Specifically, elevated neck circumference was associated with 4.72-fold higher odds of dyslipidemia (95% CI: 1.66–13.37, *p* = 0.004), breakfast skipping with 3.39-fold higher odds (95% CI: 1.67–6.88, *p* = 0.001), and HbA1c with 3.61-fold higher odds (95% CI: 1.82–7.13, *p* < 0.001). Across all models, age, sex, and elevated waist circumference were not independently associated with dyslipidemia. Model fit improved progressively with sequential adjustment, with Nagelkerke R^2^ increasing from 0.022 in Model 1 to 0.228 in Model 4.

In a sensitivity analysis addressing the nonlinear association between HbA1c and dyslipidemia, HbA1c was dichotomized at the prediabetes threshold (≥5.7%). The results were consistent with the primary analysis: elevated NC (OR: 5.41, 95% CI: 1.93–15.17, *p* = 0.001), breakfast skipping (OR: 3.25, 95% CI: 1.63–6.49, *p* = 0.001), and HbA1c ≥ 5.7% (OR: 1.80, 95% CI: 1.05–3.10, *p* = 0.033) remained independently associated with dyslipidemia. Model calibration improved (Hosmer–Lemeshow *p* = 0.704).

## 4. Discussion

This cross-sectional comparative study evaluated meal pattern characteristics and anthropometric indicators in adults with newly diagnosed dyslipidemia and normolipidemic controls. The main findings were that breakfast skipping was more common in the dyslipidemic group and remained independently associated with dyslipidemia in the fully adjusted model; elevated NC was also independently associated with dyslipidemia; WHtR category distribution differed significantly between groups; and neither BMI nor elevated WC differed significantly between groups.

In addition, elevated NC and elevated WC were both associated with a higher dyslipidemia component count, suggesting that anthropometric markers of fat distribution may relate not only to dyslipidemia status itself, but also to the burden of lipid abnormalities. Together, these findings suggest that, in adults with newly diagnosed dyslipidemia, selected fat distribution markers and behavioral factors may be more informative than conventional obesity metrics alone.

The absence of a significant BMI difference between groups is noteworthy and is consistent with evidence indicating that BMI has limited specificity for identifying cardiometabolic risk related to adverse fat distribution. Contemporary literature emphasizes that obesity-related dyslipidemia is driven not only by excess adiposity itself, but also by adipose tissue distribution and dysfunction, with characteristic alterations including elevated triglycerides and reduced HDL cholesterol [26]. In this context, the lack of a BMI difference despite marked differences in lipid parameters supports the relevance of anthropometric indicators reflecting fat distribution rather than total body mass alone.

Elevated NC was substantially more frequent in dyslipidemic participants and remained independently associated with dyslipidemia in the fully adjusted model. This finding is consistent with growing evidence supporting NC as a practical marker of upper-body adiposity with metabolic relevance beyond BMI and WC. In the Framingham Heart Study, NC was associated with triglycerides and inversely associated with HDL cholesterol even after adjustment for visceral adipose tissue and BMI, suggesting that upper-body subcutaneous fat may represent a metabolically relevant fat depot [27]. More recently, a large population-based analysis from the German National Cohort confirmed that NC was associated with multiple cardiometabolic risk factors, including lipid and glycemic traits [28]. The biological plausibility of this association is also supported by evidence that upper-body fat depots contribute importantly to circulating free fatty acid flux, a pathway linked to insulin resistance and dyslipidemia [29]. Accordingly, our findings support the view that NC may provide complementary clinical information for identifying dyslipidemia risk. However, the relatively wide confidence interval for NC indicates that this association should be interpreted cautiously and confirmed in larger samples.

Although elevated WC was not independently associated with dyslipidemia in the multivariable models, elevated WC and WHtR categories were associated with a greater dyslipidemia component burden in subgroup analyses, mainly driven by lower HDL levels. This pattern is biologically plausible and broadly consistent with evidence linking central adiposity to atherogenic lipid abnormalities, particularly reduced HDL cholesterol [30]. In addition, the significant between-group difference in WHtR category distribution supports the relevance of fat distribution markers in this population. Overall, these findings suggest that although WC did not remain independently associated with dyslipidemia after adjustment, anthropometric indicators of fat distribution still captured differences in lipid burden that were not reflected by BMI alone. The finding that elevated NC, but not elevated WC, was independently associated with dyslipidemia is noteworthy and may reflect the distinct metabolic roles of the fat depots captured by these two measurements. WC primarily reflects visceral and abdominal subcutaneous adiposity, whereas NC is considered a surrogate for upper-body subcutaneous fat, a depot that contributes substantially to circulating free fatty acid flux independently of visceral fat [29]. In a population where the majority of individuals already present with elevated WC, as in the present study, NC may capture additional metabolic variation related to ectopic lipid partitioning that is not reflected by abdominal circumference alone. This interpretation is further supported by data from the Framingham Heart Study, in which NC remained associated with triglycerides and inversely associated with HDL cholesterol even after adjustment for visceral adipose tissue and BMI [27]. Rather than diminishing the relevance of WC as a clinical tool, these findings suggest that NC and WC may provide complementary information in dyslipidemia risk assessment, and that NC may be particularly informative in populations with a high prevalence of abdominal obesity. NC measurement is simple, quick, and does not require clothing removal, which may facilitate its use as a complementary anthropometric tool in outpatient settings.

Breakfast skipping was more frequent among dyslipidemic participants and remained independently associated with dyslipidemia after adjustment for demographic, anthropometric, and glycemic variables. This finding is consistent with previous observational and longitudinal evidence linking irregular breakfast habits to unfavorable lipid profiles. A retrospective cohort study in a working population reported that lower breakfast frequency was associated with greater odds of dyslipidemia [31]. A systematic review and meta-analysis of randomized controlled trials and prospective cohort studies further supported an association between breakfast skipping and unfavorable changes in lipid profiles, particularly higher LDL cholesterol [9]. A recent meta-analysis further supported an association between breakfast skipping and dyslipidemia within the broader metabolic syndrome framework [32]. At the mechanistic level, breakfast omission has also been associated with a more unfavorable lipoprotein profile, including higher small-dense LDL cholesterol concentrations [33]. Several mechanisms may underlie this association. Breakfast omission prolongs the overnight fasting period, which may alter circadian alignment of peripheral metabolic clocks, impair morning insulin sensitivity, and increase free fatty acid mobilization from adipose tissue, thereby promoting hepatic lipogenesis and an atherogenic lipoprotein profile [8]. Additionally, compensatory overeating at subsequent meals may contribute to postprandial lipemic responses and unfavorable shifts in daily energy distribution [34]. However, the cross-sectional design of the present study precludes causal interpretation.

Interestingly, lunch skipping was significantly less common among dyslipidemic participants than among normolipidemic controls. Although this finding may appear counterintuitive, it may reflect compensatory meal redistribution: a large population-based study using NHANES data showed that adults who skipped breakfast consumed significantly more energy at lunch, suggesting that breakfast omission may lead to compensatory intake at subsequent meals rather than overall meal omission [34]. This pattern is also consistent with evidence that the timing and distribution of meals across the day may be more relevant to metabolic outcomes than the number of meals skipped per se [10]. However, this association was observed only in univariate analysis and was not included in the multivariable models, because the study was primarily designed to evaluate breakfast skipping. This finding should therefore be interpreted with caution and may warrant further investigation in studies with more detailed dietary assessment.

Dyslipidemic participants also had significantly higher fasting glucose and HbA1c levels despite the exclusion of overt diabetes, and HbA1c remained independently associated with dyslipidemia in the fully adjusted model. This finding is consistent with the known clustering of dyslipidemia and early glycemic dysregulation within broader cardiometabolic risk states. Current diagnostic standards define HbA1c values of 5.7–6.4% as prediabetes [25], and population-based data have shown that dyslipidemia prevalence increases across higher HbA1c categories, including the prediabetic range [35]. Together, these findings suggest that newly diagnosed dyslipidemia may frequently coexist with early disturbances in glycemic regulation, even in the absence of overt diabetes. HbA1c was included only in the final model as an exploratory glycemic adjustment; because glycemic markers may partly reflect metabolic processes overlapping with dyslipidemia, this model should be interpreted as a robustness analysis rather than a strictly causal adjustment model.

The hierarchical regression analysis showed that model performance improved after the addition of anthropometric indicators, breakfast skipping, and HbA1c, although the explanatory power of the final model remained modest. This is not unexpected given the multifactorial nature of dyslipidemia. From a clinical perspective, elevated NC and breakfast skipping may offer practical complementary information, because both can be assessed easily in outpatient settings. However, these findings should be interpreted cautiously and confirmed in prospective studies with more detailed dietary and lifestyle assessment.

This study has several limitations. First, its cross-sectional design precludes causal inference and does not permit determination of temporal direction. Second, the single-center outpatient setting may limit generalizability. Third, meal pattern characteristics were self-reported and may therefore be affected by recall bias and reporting inaccuracies. Moreover, meal pattern assessment was limited to skipping behavior and did not include detailed information on meal composition, portion size, timing regularity, or total energy intake. Fourth, data on diet quality, physical activity, sleep, work schedule, and other potential lifestyle confounders were not available, and residual confounding cannot be excluded. Fifth, the meal pattern questionnaire was developed specifically for this study and was not formally validated or pilot-tested; although it comprised simple factual items, the absence of established psychometric properties should be considered when interpreting the findings. Sixth, although all anthropometric measurements were performed by a single trained assessor who was blinded to lipid status, intra-observer repeatability was not formally assessed.

## 5. Conclusions

In this cross-sectional comparative study of adults without diabetes, breakfast skipping and elevated neck circumference were independently associated with newly diagnosed dyslipidemia after adjustment for demographic, anthropometric, and glycemic variables. Elevated neck circumference and waist circumference were also associated with a greater dyslipidemia component burden. These findings suggest that simple behavioral and anthropometric markers, particularly breakfast skipping and neck circumference, may help identify individuals at higher risk of dyslipidemia in outpatient settings and support early preventive assessment alongside conventional obesity measures. Prospective studies are needed to confirm these associations and clarify their temporal direction.

## Figures and Tables

**Table 1:** Clinical and anthropometric characteristics of normolipidemic and dyslipidemic participants.

Variable	Total (*n* = 257)	Normolipidemic (*n* = 120)	Dyslipidemic (*n* = 137)	*p*
Age (years)	51.22 ± 9.47	50.18 ± 10.69	52.14 ± 8.19	0.103
Female sex	166 (64.6%)	82 (68.3%)	84 (61.3%)	0.240
Glucose (mg/dL)	102.54 ± 18.39	95.31 ± 14.79	108.88 ± 18.93	**<0.001**
HbA1c (%)	5.72 ± 0.43	5.58 ± 0.44	5.84 ± 0.39	**<0.001**
Triglycerides (mg/dL)	140 (107–214)	118 (94–133)	210 (173–298)	**<0.001 ^†^**
HDL (mg/dL)	50.62 ± 12.71	61.29 ± 9.68	41.28 ± 5.80	**<0.001**
LDL (mg/dL)	129.82 ± 31.83	102.07 ± 18.10	154.13 ± 18.58	**<0.001**
BMI (kg/m^2^)	30.07 ± 5.45	29.69 ± 6.04	30.41 ± 4.87	0.296
Elevated WC	201 (78.2%)	88 (73.3%)	113 (82.5%)	0.076
Elevated NC	225 (87.5%)	94 (78.3%)	131 (95.6%)	**<0.001**
WHtR category				**0.048**
No risk	15 (5.8%)	10 (8.3%)	5 (3.6%)	
Increased risk	65 (25.3%)	36 (30.0%)	29 (21.2%)	
High risk	177 (68.9%)	74 (61.7%)	103 (75.2%)	
WHtR	0.63 ± 0.08	0.62 ± 0.09	0.64 ± 0.08	0.094

Continuous variables are presented as mean ± standard deviation or median (interquartile range), as appropriate; categorical variables are presented as *n* (%). Comparisons between groups were performed using the independent samples *t*-test, Mann–Whitney U test (^†^), chi-square test, or Fisher’s exact test, as appropriate. NC: neck circumference; WC: waist circumference; WHtR: waist-to-height ratio. Elevated NC and elevated WC were defined according to sex-specific cutoff values. WHtR was classified into predefined risk categories. Bold values indicate statistical significance (*p* < 0.05).

**Table 2:** Meal pattern characteristics of normolipidemic and dyslipidemic participants.

Variable	Total (*n* = 257)	Normolipidemic (*n* = 120)	Dyslipidemic (*n* = 137)	*p*
Main meals/day	2.36 ± 0.51	2.33 ± 0.52	2.39 ± 0.51	0.342
Snacks/day	0.91 ± 0.93	0.83 ± 0.94	0.99 ± 0.92	0.150
Breakfast skipping	60 (23.3%)	18 (15.0%)	42 (30.7%)	**0.003**
Lunch skipping	151 (58.8%)	79 (65.8%)	72 (52.6%)	**0.031**
Dinner skipping	25 (9.7%)	8 (6.7%)	17 (12.4%)	0.121
Snack skipping	227 (88.3%)	105 (87.5%)	122 (89.1%)	0.699

Continuous variables are presented as mean ± standard deviation. Continuous variables were compared using the independent samples *t*-test. Categorical variables are presented as number (percentage) and were compared using the chi-square test. Bold values indicate statistical significance (*p* < 0.05).

**Table 3:** Dyslipidemia component burden and lipid parameters across anthropometric indicators.

Anthropometric Indicator (*n* = 257)	Dyslipidemia Component Count	TG (mg/dL)	HDL (mg/dL)	LDL (mg/dL)
Elevated NC				
No	0.56 ± 1.18	132.5 (101.7–148.2)	60.4 ± 18.1	110.0 ± 39.3
Yes	1.48 ± 1.32	144.0 (108.0–221.5)	49.2 ± 11.1	132.6 ± 29.6
*p*-value	**<0.001**	0.063	**0.002**	**0.003**
Elevated WC				
No	1.02 ± 1.24	141.5 (115.7–196.0)	53.9 ± 17.5	125.0 ± 39.9
Yes	1.46 ± 1.36	140.0 (106.5–216.5)	49.7 ± 10.9	131.2 ± 29.1
*p*-value	**0.023**	0.810	**0.030**	0.285
WHtR category				
No risk	1.00 ± 1.46	137.0 (111.0–221.0)	55.9 ± 21.5	123.4 ± 48.0
Increased risk	1.08 ± 1.27	137.0 (121.1–197.5)	52.3 ± 14.4	126.3 ± 34.2
High risk	1.50 ± 1.35	146.0 (105.0–216.5)	49.6 ± 10.9	131.7 ± 29.2
*p*-value	0.051	0.955	0.081	0.365

Continuous variables are presented as mean ± standard deviation or median (Q1–Q3), as appropriate. Comparisons between two groups were performed using the independent samples *t*-test or the Mann–Whitney U test, as appropriate. Comparisons across WHtR categories were performed using one-way ANOVA or the Kruskal–Wallis test, as appropriate. NC: neck circumference; WC: waist circumference; WHtR: waist-to-height ratio. Elevated NC and elevated WC were defined according to sex-specific cutoff values. Dyslipidemia component count represents the number of abnormal lipid parameters and ranges from 0 to 3. Bold values indicate statistical significance (*p* < 0.05).

**Table 4:** Hierarchical logistic regression models for dyslipidemia.

Model	Variable	B	S.E.	Wald	*p*-Value	OR (95% CI)
Model 1 Demographic model	Constant	−1.123	0.710	2.502	0.114	—
	Age	0.022	0.013	2.751	0.097	1.02 (0.996–1.050)
	Sex (male)	0.314	0.265	1.408	0.235	1.37 (0.82–2.30)
	Model χ^2^ = 4.19				*p* = 0.123	
	Nagelkerke R^2^ = 0.022, Hosmer–Lemeshow *p* = 0.058					
	**Variable**	**B**	**S.E.**	**Wald**	***p*-value**	**OR (95% CI)**
Model 2 Anthropometric model	Constant	−2.130	0.837	6.480	0.011	—
	Age	0.008	0.015	0.288	0.591	1.01 (0.98–1.04)
	Sex (male)	0.429	0.305	1.982	0.159	1.54 (0.85–2.79)
	Elevated NC	1.636	0.497	10.859	**0.001**	**5.14 (1.94–13.60)**
	Elevated WC	0.304	0.388	0.613	0.433	1.36 (0.63–2.90)
	Model χ^2^ = 21.06				***p* < 0.001**	
	Nagelkerke R^2^ = 0.105, Hosmer–Lemeshow *p* = 0.826					
	**Variable**	**B**	**S.E.**	**Wald**	***p*-value**	**OR (95% CI)**
Model 3 Behavioral model	Constant	−2.641	0.862	9.377	0.002	—
	Age	0.009	0.015	0.322	0.570	1.01 (0.98–1.04)
	Sex (male)	0.428	0.313	1.875	0.171	1.53 (0.83–2.83)
	Elevated NC	1.797	0.518	12.016	**0.001**	**6.03 (2.18–16.65)**
	Elevated WC	0.410	0.403	1.033	0.309	1.51 (0.68–3.32)
	Breakfast skipping	1.166	0.350	11.107	**0.001**	**3.21 (1.62–6.37)**
	Model χ^2^ = 33.42				***p* < 0.001**	
	Nagelkerke R^2^ = 0.163, Hosmer–Lemeshow *p* = 0.245					
	**Variable**	**B**	**S.E.**	**Wald**	***p*-value**	**OR (95% CI)**
Model 4 Glycemic- adjusted model	Constant	−9.421	2.075	20.609	<0.001	—
	Age	0.004	0.016	0.051	0.821	1.00 (0.97–1.04)
	Sex (male)	0.461	0.321	2.053	0.152	1.59 (0.84–2.98)
	Elevated NC	1.551	0.532	8.507	**0.004**	**4.72 (1.66–13.37)**
	Elevated WC	0.291	0.415	0.491	0.484	1.34 (0.59–3.02)
	Breakfast skipping	1.219	0.362	11.351	**0.001**	**3.39 (1.67–6.88)**
	HbA1c	1.283	0.348	13.616	**<0.001**	**3.61 (1.82–7.13)**
	Model χ^2^ = 48.06				***p* < 0.001**	
	Nagelkerke R^2^ = 0.228, Hosmer–Lemeshow *p* = 0.168					

Odds ratios (ORs) are presented with 95% confidence intervals (CIs). NC: neck circumference; WC: waist circumference. Elevated NC and elevated WC were coded as 0 = No and 1 = Yes. Sex was coded as 0 = female and 1 = male. Breakfast skipping was coded as 0 = never skip and 1 = always or sometimes skip. HbA1c was entered as a continuous variable; the corresponding OR represents the change in odds per 1-percentage-point increase. Model fit was assessed using the likelihood ratio chi-square test (Model χ^2^), Nagelkerke R^2^, and the Hosmer–Lemeshow goodness-of-fit test. Multicollinearity was evaluated using variance inflation factor (VIF) values derived from an equivalent linear regression model; all VIF values were below 2, indicating no evidence of multicollinearity. Bold values indicate statistical significance (*p* < 0.05).

## Data Availability

The datasets generated and/or analyzed during the current study are not publicly available due to institutional data protection policies but are available from the corresponding author on reasonable request.

## Supplementary Materials

The following supporting information can be downloaded at: https://www.mdpi.com/article/10.3390/jcm15103734/s1, Figure S1: Participant Flow Diagram; File S1: Data Collection Form.

## Abbreviations

The following abbreviations are used in this manuscript:

TG	Triglycerides
HDL	High-density lipoprotein cholesterol
LDL	Low-density lipoprotein cholesterol
BMI	Body mass index
WC	Waist circumference
NC	Neck circumference
WHtR	Waist-to-height ratio

## References

[1] Von Krüchten R., Lorbeer R., Müller-Peltzer K., Rospleszcz S., Storz C., Askani E., Kulka C., Schuppert C., Rathmann W., Peters A. (2022). Association between adipose tissue depots and dyslipidemia: The KORA-MRI population-based study. Nutrients.

[2] Liu H., Eso A., Cook N., O’Neill H., Albarqouni L. (2024). Meal timing and anthropometric and metabolic outcomes. JAMA Netw. Open.

[3] Ha K., Song Y. (2019). Associations of meal timing and frequency with obesity and metabolic syndrome among Korean adults. Nutrients.

[4] Teixeira G.P., Guimarães K.C., Soares A.G.N., Marqueze E.C., Moreno C.R., Mota M.C., Crispim C.A. (2023). Role of chronotype in dietary intake, meal timing, and obesity: A systematic review. Nutr. Rev..

[5] St-Onge M.P., Ard J., Baskin M.L., Chiuve S.E., Johnson H.M., Kris-Etherton P., Varady K. (2017). Meal timing and frequency: Implications for cardiovascular disease prevention: A scientific statement from the American Heart Association. Circulation.

[6] Gao W., Zhe H., Ba D., Jigeer G., Liu Y., Chen S., Li Y., Sun L., Wu S., Gao X. (2025). Habitual breakfast skipping and night eating associated with unfavorable changes in lipid profiles in Chinese adults: A longitudinal analysis. Am. J. Clin. Nutr..

[7] Raji O., Kyeremah E., Sears D., St-Onge M., Makarem N. (2024). Chrononutrition and cardiometabolic health: An overview of epidemiological evidence and key future research directions. Nutrients.

[8] Dashti H.S., Makarem N., Engwall A., Gómez-Abellán P., Qian J., Brindle R.C., Bertisch S.M., Redline S., Garaulet M., Scheer F.A.J.L. (2025). Advancing chrononutrition for cardiometabolic health: A 2023 National Heart, Lung, and Blood Institute Workshop Report. J. Am. Heart Assoc..

[9] Yu J., Xia J., Xu D., Wang Y., Yin S., Lu Y., Xia H., Wang S., Sun G. (2023). Effect of skipping breakfast on cardiovascular risk factors: A grade-assessed systematic review and meta-analysis of randomized controlled trials and prospective cohort studies. Front. Endocrinol..

[10] Yong A.S.J., Koo R.W.X., Ng C.M., Lee S.W.H., Teoh S.L. (2024). Effect of meal timing and frequency on lipid profile in adults: An overview of systematic reviews and meta-analyses. Nutr. Food Sci..

[11] Hourizadeh J., Munshi R., Zeltser R., Makaryus A.N. (2024). Dietary effects of fasting on the lipid panel. Curr. Cardiol. Rev..

[12] Santos H.O., Tinsley G.M. (2024). Is breakfast consumption detrimental, unnecessary, or an opportunity for health promotion? A review of cardiometabolic outcomes and functional food choices. Diabetes Metab. Res. Rev..

[13] Ross R., Neeland I.J., Yamashita S., Shai I., Seidell J., Magni P., Santos R.D., Arsenault B., Cuevas A., Hu F.B. (2020). Waist circumference as a vital sign in clinical practice: A Consensus Statement from the IAS and ICCR Working Group on Visceral Obesity. Nat. Rev. Endocrinol..

[14] Chan V., Cao L., Wong M.M.H., Lo K., Tam W. (2024). Diagnostic Accuracy of Waist-to-Height Ratio, Waist Circumference, and Body Mass Index in Identifying Metabolic Syndrome and Its Components in Older Adults: A Systematic Review and Meta-Analysis. Curr. Dev. Nutr..

[15] Cao L., Zhou J., Chen Y., Wu Y., Wang Y., Liu T., Fu C. (2022). Effects of Body Mass Index, Waist Circumference, Waist-to-Height Ratio and Their Changes on Risks of Dyslipidemia among Chinese Adults: The Guizhou Population Health Cohort Study. Int. J. Environ. Res. Public Health.

[16] Araste A., Shadmand Foumani Moghadam M.R., Mastali M., Ganjali R., Eslami S., Khosravi M., Rezaee R., Rezvani R. (2025). Neck circumference can be a better predictor of cardiometabolic syndrome among body shape indexes and other anthropometry parameters—A cross-sectional study from Mashhad Persian Cohort. Clin. Obes..

[17] National Cholesterol Education Program (NCEP) Expert Panel on Detection, Evaluation, and Treatment of High Blood Cholesterol in Adults (Adult Treatment Panel III) (2002). Third report of the National Cholesterol Education Program (NCEP) Expert Panel on Detection, Evaluation, and Treatment of High Blood Cholesterol in Adults (Adult Treatment Panel III) final report. Circulation.

[18] Alberti K.G., Eckel R.H., Grundy S.M., Zimmet P.Z., Cleeman J.I., Donato K.A., Fruchart J.C., James W.P., Loria C.M., Smith S.C. (2009). Harmonizing the metabolic syndrome: A joint interim statement of the International Diabetes Federation Task Force on Epidemiology and Prevention; National Heart, Lung, and Blood Institute; American Heart Association; World Heart Federation; International Atherosclerosis Society; and International Association for the Study of Obesity. Circulation.

[19] Enani S., Bahijri S., Malibary M., Jambi H., Eldakhakhny B., Al-Ahmadi J., Al Raddadi R., Ajabnoor G., Boraie A., Tuomilehto J. (2020). The association between dyslipidemia, dietary habits and other lifestyle indicators among non-diabetic attendees of primary health care centers in Jeddah, Saudi Arabia. Nutrients.

[20] Alkhulaifi F., Al-Hooti S., Al-Zenki S., Alomirah H., Xiao Q., Chan W., Wu F., Darkoh C. (2024). Association of nightly fasting, meal frequency, and skipping meals with metabolic syndrome among Kuwaiti adults. Nutrients.

[21] World Health Organization (2000). Obesity: Preventing and Managing the Global Epidemic.

[22] World Health Organization (2008). Waist Circumference and Waist-Hip Ratio: Report of a WHO Expert Consultation.

[23] Ashwell M. (2009). Obesity risk: Importance of the waist-to-height ratio. Nurs. Stand..

[24] Ben-Noun L., Sohar E., Laor A. (2001). Neck circumference as a simple screening measure for identifying overweight and obese patients. Obes. Res..

[25] American Diabetes Association Professional Practice Committee (2024). Diagnosis and classification of diabetes: Standards of care in diabetes—2024. Diabetes Care.

[26] Bays H.E., Kirkpatrick C.F., Maki K.C., Toth P.P., Morgan R.T., Tondt J., Christensen S.M., Dixon D.L., Jacobson T.A. (2024). Obesity, dyslipidemia, and cardiovascular disease: A joint expert review from the Obesity Medicine Association and the National Lipid Association 2024. J. Clin. Lipidol..

[27] Preis S.R., Massaro J.M., Hoffmann U., D′Agostino R.B., Levy D., Robins S.J., Meigs J.B., Vasan R.S., O′Donnell C.J., Fox C.S. (2010). Neck circumference as a novel measure of cardiometabolic risk: The Framingham Heart Study. J. Clin. Endocrinol. Metab..

[28] Strathmann E.A., Ratjen I., Willrodt K., Enderle J., Schlesinger S., Fischer B., Weber K.S., Övermöhle C., Greiser K.H., Sedlmeier A.M. (2025). Association of neck circumference with cardiometabolic risk factors and diseases in the German National Cohort. J. Endocr. Soc..

[29] Nielsen S., Jensen M.D. (2024). Insulin regulation of regional lipolysis in upper-body obese and lean humans. JCI Insight.

[30] Riutord-Sbert P., Tárraga López P.J., López-González Á.A., Coll Campayo I., Busquets-Cortés C., Ramírez Manent J.I. (2025). Determinants of atherogenic dyslipidemia and lipid ratios: Associations with sociodemographic profile, lifestyle, and social isolation in Spanish workers. J. Clin. Med..

[31] Li Q.-M., Wu C.-K., Ma P.-C., Cui H., Li R.-N., Hong C., Zeng L., Liao S.-W., Xiao L.-S., Liu L. (2022). Breakfast consumption frequency is associated with dyslipidemia: A retrospective cohort study of a working population. Lipids Health Dis..

[32] Yang B., Lian L., Xing K., Cen Y., Zhao Y., Zhang Y. (2025). Association of skipping breakfast with metabolic syndrome and its components: A systematic review and meta-analysis of observational studies. Nutrients.

[33] Arimoto M., Yamamoto Y., Imaoka W., Kuroshima T., Toragai R., Nakamura M., Ito Y., Ai M. (2023). Small dense low-density lipoprotein cholesterol levels in breakfast skippers and staple foods skippers. J. Atheroscler. Thromb..

[34] Zeballos E., Todd J.E. (2020). The effects of skipping a meal on daily energy intake and diet quality. Public Health Nutr..

[35] Kang P., Kim K.Y., Shin H.Y. (2024). Association between dyslipidemia and glycated hemoglobin in a population-based study. Metabolites.

