# Supplementary material for: Breakfast Skipping and Elevated Neck Circumference Are Independently Associated with Newly Diagnosed Dyslipidemia in Adults Without Diabetes
Source: J Clin Med. 2026 May 13;15(10):3734. doi: 10.3390/jcm15103734 (PMC13207040; doi:10.3390/jcm15103734)
Supplement: Supplementary file 1 [file jcm-15-03734-s001.zip › Supplementary File S1.pdf]

Supplementary File S1. Data Collection Form

1. **Age: …………………………… years**
2. **Sex:** □ Male □ Female
3. **Dyslipidemia status:** □ Newly diagnosed dyslipidemia □ No (Normolipidemia)
4. **Antropometric Meuserements**

   Height: …………………. cm

   Weight: …………………… kg

   Waist circumference: ………….. cm

   Neck circumference: ……………. cm
5. **How many meals do you usually consume per day?**

   Main meals (breakfast, lunch, dinner): …………………… per day

   Snacks: …………………… per day
6. **Please indicate how often you skip the following meals:**

Meal Type	Always skip	Sometimes skip	Never skip
**Breakfast**	□	□	□
**Lunch**	□	□	□
**Dinner**	□	□	□
**Snacks**	□	□	□

7. **Biochemical Parameters (Routine Laboratory Results)**

   Triglycerides (TG): ………………………… mg/dL

   LDL cholesterol (LDL-C): ………………………… mg/dL

   HDL cholesterol (HDL-C): ………………………… mg/dL

   Fasting glucose: ………………………… mg/dL

   HbA1c: ………………………… %
