# Supplementary figures and images for: Breakfast Skipping and Elevated Neck Circumference Are Independently Associated with Newly Diagnosed Dyslipidemia in Adults Without Diabetes
Source: J Clin Med. 2026 May 13;15(10):3734. doi: 10.3390/jcm15103734 (PMC13207040; doi:10.3390/jcm15103734)

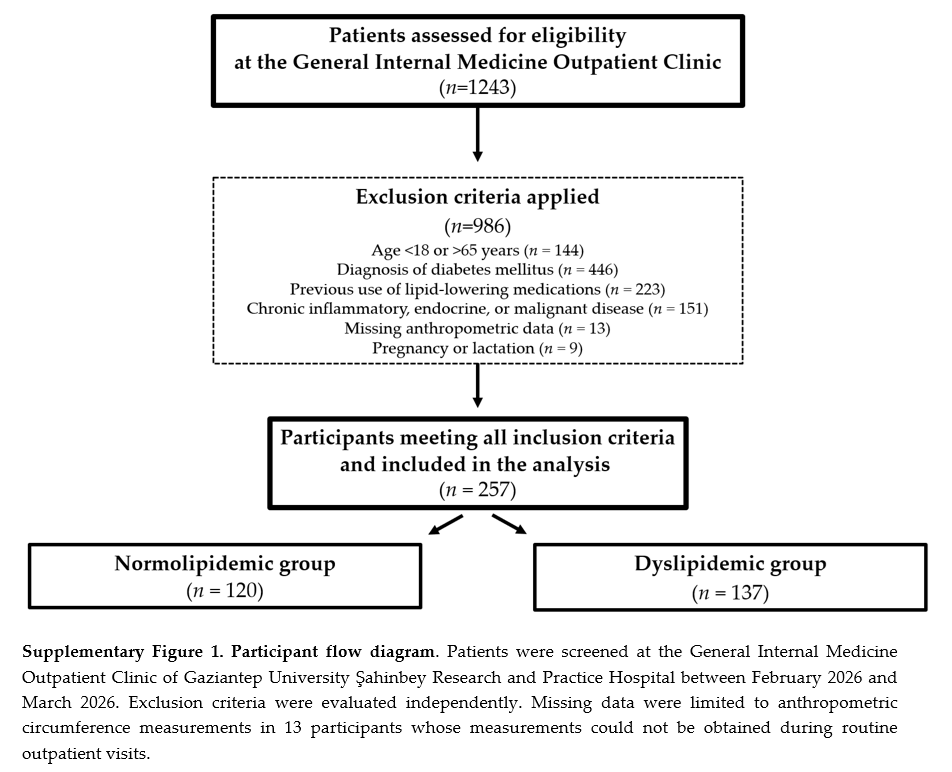

Supplement: Supplementary file 1 [file jcm-15-03734-s001.zip › Figures S1.png]
